# Spatial and temporal variation of CBPI and leakage of heavy metals from cigarette butts into the urban environment

**DOI:** 10.1038/s41598-023-28340-6

**Published:** 2023-01-25

**Authors:** Khadijeh Darabi, Ghasem Hassani, Navid Alinejad, Ahmad Badeenezhad

**Affiliations:** 1 Department of Environmental Health Engineering, Shiraz University of Medical Sciences, Shiraz, Iran; 2 Department of Environmental Health Engineering, Faculty of Health, Yasuj University of Medical Sciences, Yasuj, Iran; 3 Department of Public Health, Fasa University of Medical Sciences, Fasa, Iran; 4 Department of Environmental Health Engineering, School of Medical Sciences, Behbahan Faculty of Medical Sciences, Behbahan, Iran

**Keywords:** Environmental sciences, Environmental social sciences

## Abstract

More than 5 trillion cigarettes are produced in the world every year. This hazardous waste is one of the most important litter in the environment. The purpose of this study was to investigate the density and dispersion of cigarette butts in the urban environment and to estimate the leakage of pollution from it to the environment. For this purpose, the cigarette butt pollution index was used in 14 locations. Observations were made during a year and once a month for each location. The study of the locations was done with the same conditions on weekend and working days. The amount of heavy metal leakage was estimated based on the average weight of cigarette butts and the ratio of metal leakage in different weather conditions. The results showed that the annual average of index for the studied locations was 1.36 (SD 0.11) to 10.6 (SD 1.23). Based on this, 28.5% of the locations were in the low pollution status and 42.8% were in the significant pollution status and worse. On average, the index on weekend decreased by 32.3 percent in all locations, and the average index of the studied locations in spring and summer was 26.2 percent higher than in autumn and winter. The average leakage of heavy metals including chromium, cadmium, zinc, lead, copper, and nickel from littered cigarette butts in commercial, residential, and recreational areas was estimated to be 0.27, 0.079, and 0.17 µg per square meter, respectively. Cigarette butt is one of the most abundant litter in the studied area, which is the source of many pollutants, including heavy metals. This hazardous waste is a serious threat to the urban environment.

## Introduction

Cigarette butts are one of the common litter in the environment^1^. In fact, due to the widespread consumption of filtered cigarettes, a large number of cigarette butts are produced annually in the world, which is about 4.5 billion^2^. One of the characteristics of this waste is its disposal method by smokers. Most smokers do not dispose of cigarette butts in the trash bins and littering this hazardous waste after smoking. For this reason, cigarette butts are one of the most important litter in urban environments, because of its dispersion in the environment and its long duration, the leakage of pollutants from it creates a risk for organisms^3^. A large number of littered cigarette butts in urban environments and public places such as beaches has negative effects on organisms and creates an undesirable view in tourist areas^4^. Sporadic presence of littered cigarette butts in the urban environment has caused the reduction of its collection efficiency, so cigarette butt littering is a serious challenge in urban waste management^5,6^.

Pollution leakage is one of the main problems in cigarette butt management. The pollution in cigarette smoke trapped in the cigarette filter during smoking and causes the cigarette butt to contain various pollutants^7,8^. Heavy metals are one of the known pollutants in cigarette butts that leak into the environment and can enter the food chain^9^. Lead, cadmium, copper, nickel, zinc, and chromium are the most abundant metals detected in cigarette butts^10^. In addition to heavy metals, various pollutants have been identified in cigarette butts, including organic compounds, PAHs, and nicotine^11^. The results of studies have shown that these pollutants leak from littered cigarette butts and pollute the environment. The leakage of these pollutants depends on its initial concentration in the cigarette butt and environmental conditions such as humidity^12^.

Pollutant leakage from cigarette butts causes contamination of water and soil resources, and the toxicity of cigarette butts on various organisms has been proven in many cases^13^. For example, the toxicity of cigarette butts on insects such as Aedes albopictus and Aedes Aegypti causing complications such as egg deposition, disturbance in developmental larvae stages and increased death rate^14,15^. Also, cigarette butt toxicity has caused changes in eye density and heart rate in medakaembryos^16^. In other studies, the toxic effects of cigarette butts on organisms such as Xenopus laevis embryos, Atherinops affinis, and Pimephales promelas have been reported^17,18^. In addition to the toxicity for various organisms, pollutant leakage from cigarette butts can cause pollute of water sources and increase the concentration of pollutants in landfills leachate. For example, it has been reported that an increase of 1% of cigarette butts in the landfilled waste caused a 3.7% increase in the concentration of some metals in the leachate^7^. The possibility of ingestion of cigarette butts by pets and infants, as well as the possibility of fire, are other consequences of cigarette butts littering^19^.

This study was conducted with the aim of evaluating the amount of littered cigarette butts in the urban environment, and an attempt was made to analyze the obtained results using the cigarette butt pollution index (CBPI). The purpose of using this index was to compare pollution in the studied areas. Also, in this study, the rate of leakage of heavy metals from littered cigarette butts was estimated in different weather conditions in order to use the obtained results to evaluate the annual leakage of heavy metals from cigarette butts to the urban environment.

## Method

**Figure 1:**
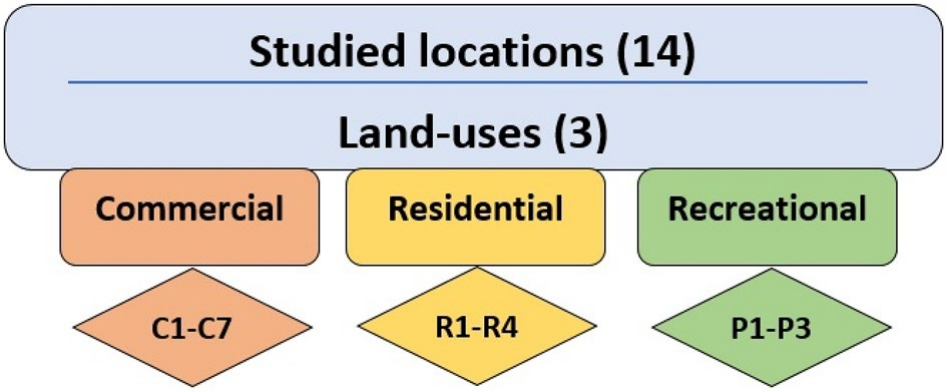
Classification of studied locations.

This study was conducted in 14 locations in Behbahan city, Iran. Based on the proposed method in previous studies, the studied locations were selected from different land-uses, including residential, commercial, and recreational^20^. As shown in Fig. 1, the studied locations included four residential locations, seven commercial locations, and three recreational locations. Littered cigarette butts were counted and due to the influence of environmental factors such as humidity on their weight, weight measurement was omitted^21^. Considering the effect of temporal variation in the density of littered cigarette butts, the survey of the locations was carried out during one year and at two times, working days and weekends for each location^22^. Also, for reducing the impact of cleaning activities on the density of littered cigarette butts, the investigation of locations and counting of littered cigarette butts were carried out in the evening hours^3^.

The interpretation of the status of the studied areas was done using the CBPI. The interpret the local status based on its results is shown in Table 1. The coefficient E used in the formula for each location was chosen based on the criteria proposed by Torkashvand et al.^23^.

1. Density of cigarette butt = $\frac{{{\text{Number}}}}{{{\text{Lenght }}\;{\text{(meter) }} \times {\text{Width }}\;({\text{meter}})}}$
2. CBPI = Density of cigarette butt × E

**Table 1:** CBPI status categories^23^.

Calculated index	Status
*Interpretation*	
≤ 1	Very low pollution
1.1–2.5	Low pollution
2.6–5	Pollution
5.1–7.5	Significant pollution
7.6–10	High pollution
> 10	Sever pollution

This index can compare the state of pollution in each location in six different modes. This index was calculated for the average density of littered cigarette butts on working days and weekends, as well as for the average of hot season and cold season in each of the studied locations. Because the amount of rainfall in the country is higher in the cold season, and due to the increase in the amount of leakage from cigarette butts in higher humidity, based on the proposed model by Torkashvand et al.^23^, the E coefficient will be higher and it is expected that the CBPI in the cold and hot seasons will be different.

The estimation of heavy metals leaked from littered cigarette butts was done using the results of previous studies. As shown in Table 2, the leakage rate of heavy metals from littered cigarette butts is different. In this study, using the amount of heavy metals measured in littered cigarette butts by Farzadkia et al.^24^, the average leakage of each metal per gram of littered cigarette butt was considered. The annual leakage rate of heavy metals in the studied locations was estimated based on the density of cigarette butts in each location under different climatic conditions in the year and considering the average expected leakage for each metal from each gram of cigarette butt. Low access point includes places that are not easy to clean, such as tree pits. Other places, such as the sidewalk, which are easy to clean, are in the easy cleanup point group.

**Table 2:** Leakage of each metal from littered cigarette butt^24^.

	Chromium	Cadmium	Copper	Zinc	Lead	Nickel
*Warm season*						
Easy cleanup point	0.05	0.005	0.65	1.24	0.23	0.08
Low access point	0.13	0.029	2.44	2.96	0.43	0.16
*Cold season*						
Easy cleanup point	0.2	0.033	3.08	2.23	0.44	0.17
Low access point	0.29	0.058	5.27	5.91	0.89	0.28

## Results and discussion

The results showed that the density of littered cigarette butt was different in the studied areas. The lowest annual average density of lice was 0.06 number/m^2^ and the highest annual average was 0.53 littered cigarette butt/m^2^. On average, 0.23 littered cigarette butt/m^2^ were observed in the fourteen studied locations. As shown in Table 3, the difference in the density of littered cigarette butt in the studied locations was 0.52 number/m^2^. Also, the average density of littered cigarette butt was different in warm months compared to cold months in all studied locations, but the difference ratio was not the same for different locations. Based on this, the highest seasonal difference of littered cigarette butt density was observed in P1, which was equal to 50%, while the lowest seasonal difference of littered cigarette butt density, equal to 7.2%, was observed in C3. On average, in the studied locations, the density of littered cigarette butt in the warm season was 26.2% different than in the cold season. The results showed that the average annual density of littered cigarette butts in residential, commercial, and recreational areas was 0.1, 0.38, and 0.07 number/m^2^, respectively.

**Table 3:** Density of littered cigarette butt in locations (number/m^2^).

	R1	C1	C2	R2	P1	C3	C4	C5	P2	R3	C6	P3	R4	C7
W_A_	0.13	0.47	0.41	0.09	0.08	0.55	0.33	0.26	0.10	0.11	0.39	0.11	0.14	0.48
C_A_	0.09	0.39	0.35	0.07	0.04	0.51	0.25	0.22	0.08	0.09	0.27	0.03	0.12	0.44
A_A_	0.11	0.43	0.38	0.08	0.06	0.53	0.29	0.24	0.09	0.10	0.33	0.07	0.13	0.46

W_A_ = Average in warm season, C_A_ = Average in cold season, A_A_ = Annual average.

**Figure 2:**
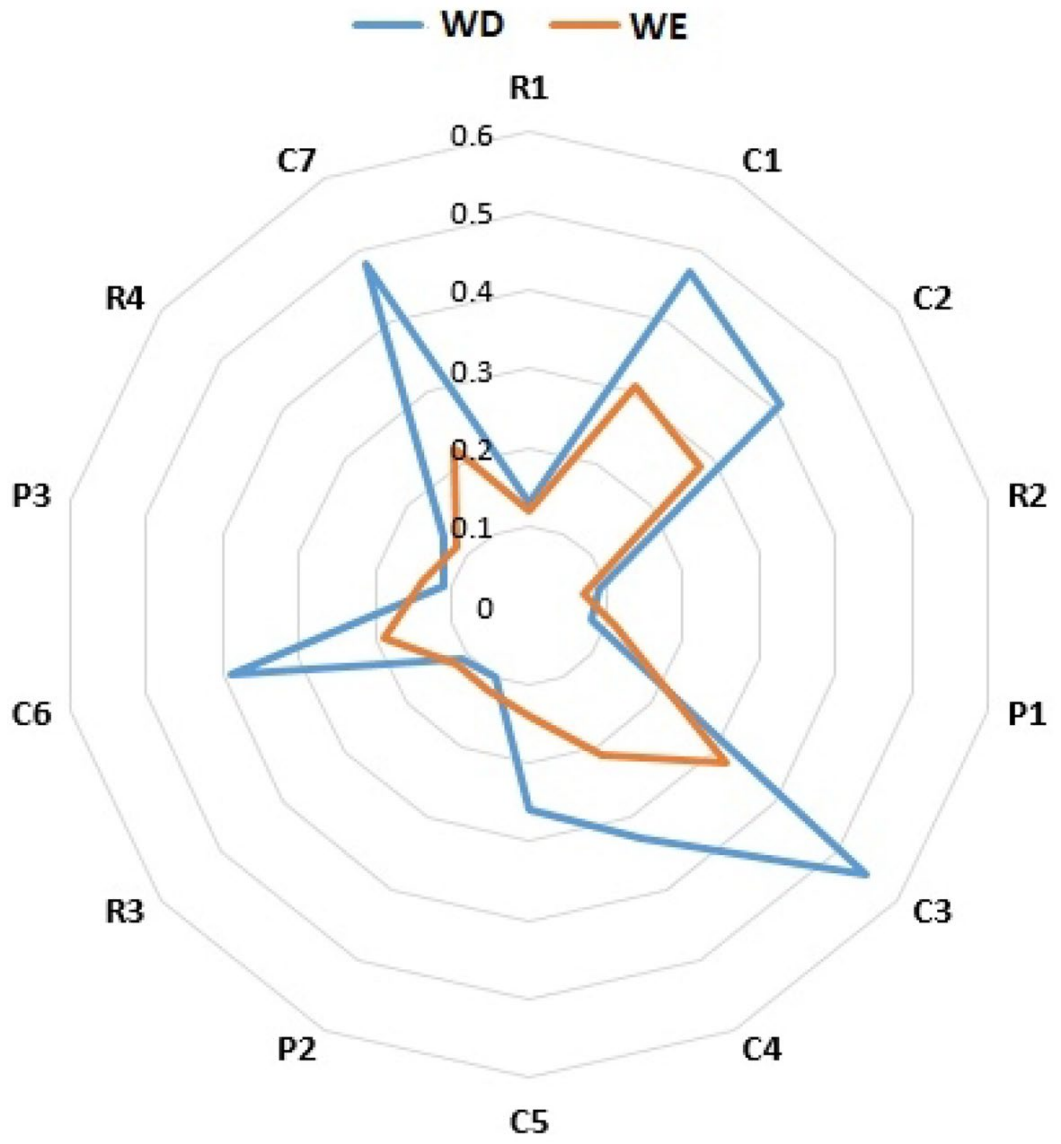
Daily variation of littered cigarette butt density in locations (WD = Working days, WE = Weekend).

In addition to seasonal variation of littered cigarette butt, daily variation of littered cigarette butt was also observed in all locations. As shown in Fig. 2, the trend of daily variation of littered cigarette butt was vary in different locations. The results showed that in 22% of the studied locations, the density of littered cigarette butt was higher on weekends than on working days, but in 78% of the studied locations, the density of littered cigarette butt was higher on working days than on weekends. On average, this variation was equal to 58.9% in locations where the density of littered cigarette butt was higher on working days, and it was equal to 27% in locations where the density of littered cigarette butt was higher on weekend. The lowest daily variation of littered cigarette butt density was observed in R3, which was equivalent to 9.1%, and the highest variation of littered cigarette butt density was observed in C7, which was equivalent to 118%.

The main reason for the pollution of the studied locations is the behavior of smokers. In different countries, most smokers do not dispose cigarette butt in the trash bins^25^. These conditions, which were also observed in this study, make littered cigarette butt one of the main components of litter in urban environments^26^. The results of researches conducted in the world have shown that littered cigarette butt has a significant share of the total litter in public areas and urban environment. Researches in this field can be divided into two categories: researches that have investigated litter and mentioned the density of littered cigarette butt in the study area (category 1), and researches that have only investigated littered cigarette butt in urban and public environments (category 1). The comparison of the results of some of these studies is mention in Table 4. This comparison shows that the density of littered cigarette butt in different study areas is not the same, which is consistent with the results of our study. Although most smokers in the world littering the cigarette butts and this is a common phenomenon all over the world^1,2^, but the density of littered cigarette butt in different countries and cities as well as in different areas of a city is influenced by various factors.

**Table 4:** Results of some research on litter and littered cigarette butt.

Study location	Main results	References
Madrid, Spain (category 2)	Spatial variations in cigarette butt density were observed in Madrid The cigarette butt density was highest around hospitality venues and public transportation stops Central locations Littered cigarette butts pollution was more in the central areas of the city than in the peripheral ones	^3^
German and Lithuanian (category 1)	The number of littered cigarette butts was different in the two countries, but their proportion in the composition of litter was similar The number of littered cigarette butts was directly related to the number of visitors on the beaches The use of ballot bin could not prevent the cigarette butt pollution of the beaches	^4^
Berlin, Germany (category 2)	The average of littered cigarette butts was 2.7 number/m^2^ Seasonal variation in the density of littered cigarette butts was not observed Nicotine leaches quickly from the cigarette butt and is known to be a serious threat to water resources	^12^
Jiroft, Iran (category 2)	Temporal variation was observed in density of littered cigarette butt The density of littered cigarette butts was higher on working days and in summer Based on the CBPI, 25% of the studied areas were in high pollution status	^22^
Mar del Plata, Argentina (category 1)	33% of all litter were littered cigarette butts, which was the highest amount among all types of litter The land-use was effective in the number of litter, including littered cigarette butts. The most litter was observed in industrial areas	^26^

One of the important factors that affect the density of littered cigarette butt is land-use^23^. As it was observed in the results of this study, the density of littered cigarette butt is varying in different land-uses. Gholami et al.^20^ by study of litter in Qazvin city pointed out this issue and stated that land-use has an important effect on the density of litter, including littered cigarette butt. The effect of land-use on population density is the most important factor that causes the variation of littered cigarette butt density in different land-uses. Places with more population will have more density of littered cigarette butt, which in our study included commercial land-uses. The reason for this phenomenon is the increase in the number of smokers in streets, which causes the density of littered cigarette butt to increase^23^. The quality of cleanup service in different parts of the city is another factor that causes variation in the density of littered cigarette butt in different parts of the city^3^. Valiente et al.^3^ by study of the Madrid pointed out the effect of the quality of cleanup on the density of littered cigarette butt because they observed that the density of littered cigarette butt was lower in places of the city where the quality of cleanup was more favorable. However, in our study, the effect of this factor can be ignored because, as other researches in Iran have shown, the quality of cleanup services was the same in all of the city^20^. But the difference between the number of two groups of points in different areas of the cities causes the variation of littered cigarette butt density. The first points are the places where the cigarette butts are more likely to be littered. Cigarette sales and use centers have been introduced as one of these points^2^. Therefore, in places where the number of cigarette sales centers is more, the possibility of littering cigarette butts is more likely. This factor can be one of the reasons for the higher density of littered cigarette butt in the commercial areas in this study. The second points are the places where the durability of litter, including littered cigarette butt, is longer. Points with limited access, such as tree pits and runoff collection channels, increase the durability of litter. Due to the reduction in the efficiency of the cleanup system in these places, the density of litter, including littered cigarette butt, will increase daily^23^.

**Figure 3:**
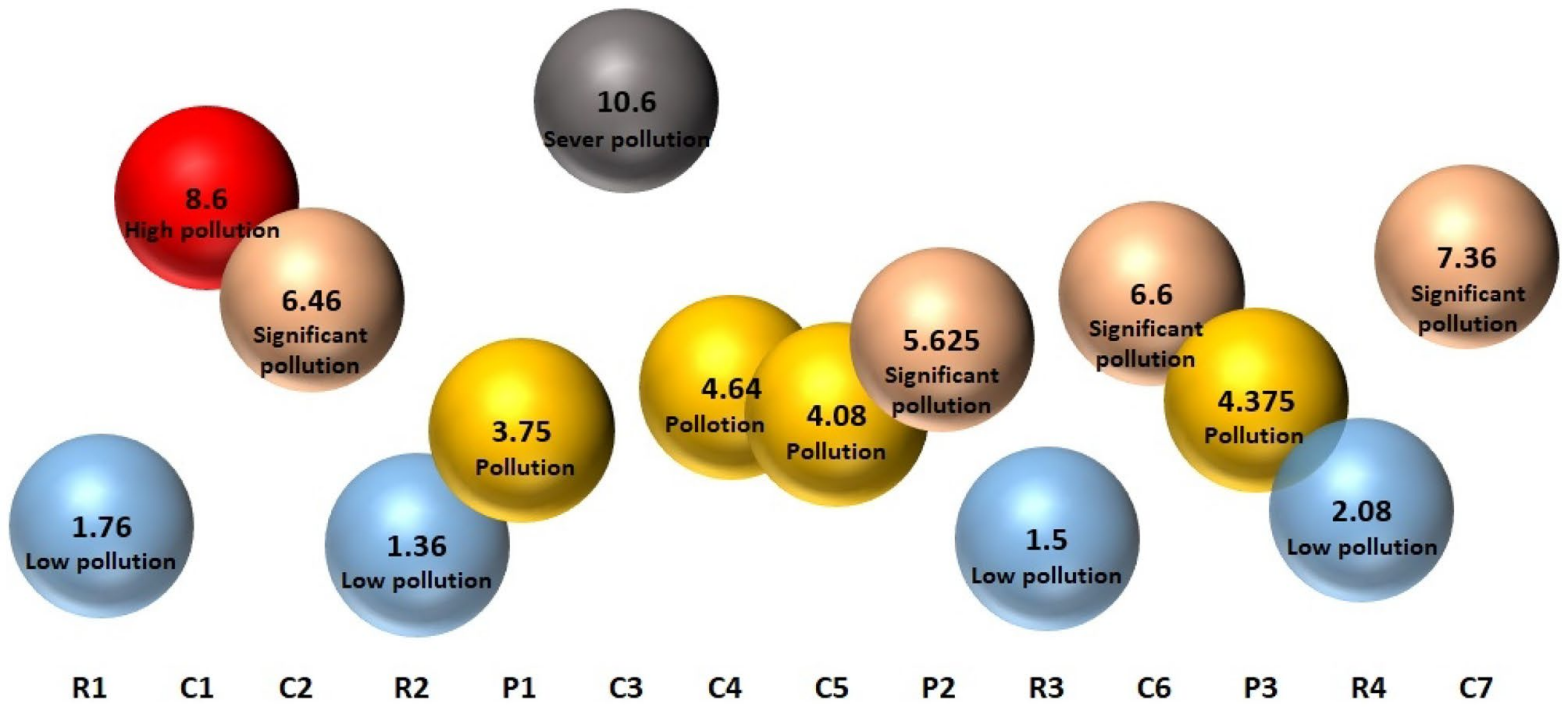
Calculated CBPI for studied locations.

The calculated cigarette butt pollution index for each location are shown in Fig. 3. The CBPI of the studied locations was vary according to the change in the density of littered cigarette butt in different locations. The highest index calculated in C3 by 10.6, which showed this location in the sever pollution status. The index calculated for R2 was equal to 1.36, which showed this location in a low pollution status and was the lowest index among the studied locations. The average index for all the studied locations was calculated as 4.91, which showed the pollution situation. According to the results, 71.4% of the studied locations were in pollution status or worse, and 28.5% of the locations were in low pollution status. The reason affecting the CBPI for each location can be classified into two groups. The first group are the factors that are effective in the density of the littered cigarette butt, and the second group are the factors that are effective in the persistence and leakage of the pollutant from the littered cigarette butt^23^. As mentioned before, factors such as population density, land-use, low access points for cleanup, and anti-littering laws are effective in the density of littered cigarette butt in different locations^3,22,23^. These factors directly increase the density of littered cigarette butt and will increase the index proportionally. On the other hand, factors such as humidity, which are effective in the amount of pollutant leakage from the littered cigarette butt, as well as factors such as the type of soil and the distance of the underground water, which are effective in the penetration of pollution leaked from the littered cigarette butt, are also effective in increasing the index due to the positive effect on the E coefficient^23,27^.

In addition to the problems caused by the density of littered cigarette butt in urban environments in terms of creating undesirable landscapes, an important consequence of these hazardous wastes is the leakage of pollutants such as heavy metals. The results of estimation of heavy metal leakage from littered cigarette butt in the studied locations are shown in Tables 5 and 6. Zinc had the highest leakage compared to other metals and cadmium had the lowest leakage among metals. On average, it was estimated that 18 µg of different metals would leak from littered cigarette butt in each square meter of the city. The reasons for the variation in the concentration of different metal leakage from littered cigarette butt include the difference in the concentration of metals in the cigarette butt, the difference in the ratio of leakage to the total concentration of different metals, and the difference in the rate of leakage of different metals^28^. In general, the concentration of various pollutants, including heavy metals, is not the same in littered cigarette butt. Due to the difference in pollutant concentration in the four main sources of pollutants in cigarette smoke, including the type of tobacco, tobacco cultivation soil, chemicals such as pesticides used in tobacco cultivation, and different additives in different cigarette factories, cigarette butts from cigarette brands contain different pollutant concentration^2^. This condition has an effect on the difference in the amount of heavy metal leakage from the cigarette butt. In addition, the difference in the smoking behavior is effective in the amount of cigarette smoke passing through the filter and the concentration of trapped pollutants in the cigarette butt^24^. Therefore, according to the difference in the potential for the presence of pollutants in cigarette smoke and the difference in the amount of cigarette smoke passing through the filter, the concentration of pollutants, including heavy metals, in cigarette butts of different brands and even cigarette butts resulting from consumption of the same brand will be different.

**Table 5:** Estimation of heavy metal leakage in warm season (μg/m^2^).

	Residential				Commercial							Recreational		
	R1	R2	R3	R4	C1	C2	C3	C4	C5	C6	C7	P1	P2	P3
Concentration of leaked metals	0.087	0.060	0.074	0.094	0.31	0.27	0.37	0.22	0.17	0.26	0.32	0.14	0.18	0.20
Average in land-use	0.079				0.27							0.17

**Table 6:** Estimation of heavy metal leakage in cold season (μg/m^2^).

	Residential				Commercial							Recreational		
	R1	R2	R3	R4	C1	C2	C3	C4	C5	C6	C7	P1	P2	P3
Concentration of leaked metals	0.23	0.16	0.20	0.25	0.86	0.75	1.01	0.60	0.48	0.71	0.88	0.14	0.18	0.20
Average in land-use	0.21				0.75							0.17		

Leakage of heavy metals from littered cigarette butt is important from two aspects. First, the leakage of metals and other trapped pollutants in the filter causes complications in various organisms, even plants^29,30^. In fact, cigarette butts as a hazardous waste can have an adverse effect on the growth process of organisms and plants^31^. In some studies, even the death of organisms exposed to cigarette butts has been reported^32^. On the other hand, in addition to the adverse effects of cigarette butts on the growth of organisms and increasing their death rate, changes in natural behaviors are known as an important effect of cigarette butts on organisms. For example, exposure to cigarette butts has disrupted the natural defensive behavior of a type of mouse against predators such as cats and snakes^33^. Therefore, littered cigarette butt in the environment can affect the health of organisms both directly and indirectly. Second, cigarette butts increase the risk of waste management and the costs of pollutant emission control in its various stages. For example, the emission of polluting gases is a serious concern in using the incineration for managing cigarette butts^5^. Also, the presence of cigarette butts in landfilled municipal solid waste will increase pollutants, including heavy metals, in landfill leachate^7^. The increase in landfill leachate pollution will increase the costs of leachate treatment and management. Leakage of heavy metals from cigarette butts varies according to its durability in the urban environment^24^. Accumulation of littered cigarette butts in low access places increases their durability in the urban environment. In this study, the amount of metal leakage was estimated by assuming four different scenarios. The first scenario was defined for places without low access point. In the second scenario, it was assumed that ten percent of cigarette butts were located in low access point. This ratio was assumed to be 15 and 25 percent for the third and fourth scenarios, respectively. The results of the estimations for each scenario in different land-uses are shown in Table 7.

**Table 7:** Leakage of heavy metals in scenarios (μg/m^2^).

	S1	S2	S3	S4
Rw	0.079	0.092	0.098	0.111
Rc	0.21	0.231	0.251	0.263
Cw	0.27	0.312	0.334	0.375
Cc	0.75	0.826	0.864	0.941
Pw	0.17	0.187	0.196	0.213
Pc	0.17	0.187	0.196	0.213

Rw = Residential areas in warm season, Rc = Residential areas in cold season, Cw = Commercial areas in warm season, Cc = Commercial areas in cold season, Pw = Recreational areas in warm season, Pc = Recreational areas in cold season.

As the results of this study showed, cigarette butts are a hazardous waste that is widely littered in the urban environment and public areas. Considering the environmental and health consequences of this hazardous waste, finding a solution to improve the situation is essential. The littering of cigarette butts by smokers has created several problems for the management of this hazardous waste. The first problem in littered cigarette butt management is collecting. The results of several studies have shown that the current methods of cleanup have little efficiency in littered cigarette butt collection, especially in environments such as the beach^1^. Due to the leakage of many pollutants, including heavy metals from littered cigarette butt, the lack of quick and high-efficiency collection has caused this waste to be known as one of the important sources of diffuse leakage of pollution in urban and public environments. Therefore, there is a need to define methods to control cigarette butt littering by smokers. Torkashvand and Farzadkia^34^ introduced the control methods of cigarette butt discharge. Based on this, educating smokers about the negative consequences of cigarette butt littering can be a priority.

Considering the pollutant emission in the form of leachate or gas in incineration and landfill methods, recycling has been introduced as a suitable solution for cigarette butts management^34^. Using cigarette butts to produce functional products can lead to the control of trapped pollutants in cigarette filters^5^. In the different methods of recycling cigarette butts, two general mechanisms can be seen to control the trapped pollutants in the cigarette filter. In the first mechanism, according to the recycling process, cigarette butts are used completely without processing in the production of products such as bricks^34^. In this way, the trapped pollutants in the cigarette butt will be encapsulated in the final product. In the second mechanism, various pollutants, including heavy metals, are separated from the cigarette butt by methods such as washing with water, chemical solvent or acid. After washing, this solution containing the pollutant can be used as a practical product^34^ or should be treated as a wastewater. Therefore, in order to safely control the leakage of heavy metals and other pollutants from cigarette butts, the littering control methods must first be implemented, and then cigarette butts must be managed as a hazardous waste separately from other solid wastes with the aim of separation and treatment the pollutants.

Although in this study, the spatial and temporal variation in the density of littered cigarette butts in the urban environment and the amount of heavy metal leakage from it were investigated, but a limitation of this study was that other pollutants were not investigated. It is suggested to investigate the leakage of other pollutants from littered cigarette butts in the urban environment in future studies.

## Conclusion

The density of littered cigarette butt in the urban environment and leakage of metals from them were evaluated. The cigarette butt pollution index showed that 75% of the studied areas were in the pollution status or worse. Land-use had a significant effect on the density of littered cigarette butt. The low efficiency of cleanup in the urban environment and the littering of cigarette butts by most smokers has caused an increase in the cigarette butt pollution index. These conditions and the potential for leaking pollutants such as heavy metals have made cigarette butts a serious threat to cities and the environment. Preventing the cigarette butt littering, increasing the efficiency of the cleanup system, and controlling the leakage of various pollutants from cigarette butts, including heavy metals, should be considered in urban planning and waste management.

## Data Availability

All data generated or analyzed during this study are included in this published article.
